# Glucocorticoid Treatment Leads to Aberrant Ion and Macromolecular Transport in Regenerating Zebrafish Fins

**DOI:** 10.3389/fendo.2019.00674

**Published:** 2019-10-04

**Authors:** Johannes R. Schmidt, Karina Geurtzen, Martin von Bergen, Kristin Schubert, Franziska Knopf

**Affiliations:** ^1^Department of Molecular Systems Biology, Helmholtz Centre for Environmental Research GmbH—UFZ, Leipzig, Germany; ^2^CRTD—Center for Regenerative Therapies Dresden, Technische Universität (TU) Dresden, Dresden, Germany; ^3^Institute of Biochemistry, Faculty of Life Sciences, University of Leipzig, Leipzig, Germany; ^4^Center for Healthy Aging, Technische Universität (TU) Dresden, Dresden, Germany

**Keywords:** proteome, zebrafish, fin regeneration, glucocorticoid, ATPase, lysosome

## Abstract

Long-term glucocorticoid administration in patients undergoing immunosuppressive and anti-inflammatory treatment is accompanied by impaired bone formation and increased fracture risk. Furthermore, glucocorticoid treatment can lead to impaired wound healing and altered cell metabolism. Recently, we showed that exposure of zebrafish to the glucocorticoid prednisolone during fin regeneration impacts negatively on the length, bone formation, and osteoblast function of the regenerate. The underlying cellular and molecular mechanisms of impairment, however, remain incompletely understood. In order to further elucidate the anti-regenerative effects of continued glucocorticoid exposure on fin tissues, we performed proteome profiling of fin regenerates undergoing prednisolone treatment, in addition to profiling of homeostatic fin tissue and fins undergoing undisturbed regeneration. By using LC-MS (liquid chromatography-mass spectrometry) we identified more than 6,000 proteins across all tissue samples. In agreement with previous reports, fin amputation induces changes in chromatin structure and extracellular matrix (ECM) composition within the tissue. Notably, prednisolone treatment leads to impaired expression of selected ECM components in the fin regenerate. Moreover, the function of ion transporting ATPases and other proteins involved in macromolecule and vesicular transport mechanisms of the cell appears to be altered by prednisolone treatment. In particular, acidification of membrane-enclosed organelles such as lysosomes is inhibited. Taken together, our data indicate that continued synthetic glucocorticoid exposure in zebrafish deteriorates cellular trafficking processes in the regenerating fin, which interferes with appropriate tissue restoration upon injury.

## Introduction

Despite bone being a highly regenerative tissue it can be permanently lost due to severe trauma and as a result of disease. In zebrafish, which are capable of complex organ regeneration, intramembranous bones of the fins and skull regenerate rapidly even after profound loss. Fin regeneration, a widely studied phenomenon of adult tissue re-growth upon fin resection (amputation) is driven by a tissue referred to as the blastema, which arises from mature cells undergoing dedifferentiation and a pool of reserved progenitor cells (1–3). The fin regenerate, being composed of different tissues (epidermis, bone, mesenchyme, blood vessels, nerves) is a structure of modest complexity which, together with the fact that it is easy to access and monitor, makes it a valuable system to study bone formation, wound healing, and cell metabolism.

Fractures in bone often occur as a result of falls and traffic accidents. Their incidence and severity, however, strongly depend on the patient's bone integrity which varies with age and general health status. Bone fragility is significantly increased during treatment with glucocorticoids, powerful drugs used in anti-inflammatory and immunosuppressive therapy, for instance to treat asthma, rheumatoid arthritis, or inflammatory bowel disease (4, 5). Despite their unmistakable advantages, therapeutic treatment with glucocorticoids causes metabolic alterations which can lead to insulin resistance, and other problems, e.g., high blood pressure (6, 7). Treatment induced bone fragility partly results from direct effects of excess glucocorticoids on bone forming osteoblasts, which show reduced proliferation (8, 9). In addition, pro-osteoclastogenic effects and changes in other organ systems may contribute to glucocorticoid-induced bone loss (5). Furthermore, bone fragility is likely to be substantiated by metabolic aberrations.

Liquid chromatography-mass spectrometry (LC-MS) based proteomics represents an informative approach to identifying and quantifying thousands of proteins in a short time. Due to the depth of analysis, affected protein groups and pathways can be comprehensively studied, providing insight into molecular mechanisms underlying complex tissue function. Thus, LC-MS based proteomics is a suitable method for profiling molecular changes within a regenerating tissue such as the fin after amputation. To date, several studies have made use of LC-MS to study the dynamics of fin regeneration and protein abundance in homeostatic caudal fin tissue (10–14). Singh et al. (14) and Rabinowitz et al. (12) used label-free proteomics in uninjured zebrafish fins, and identified 417 and 3,096 proteins, respectively. Saxena et al. (13) used difference gel electrophoresis (DIGE) based labeling with subsequent proteomics in regenerating zebrafish fins and identified 90 proteins with significantly altered abundance in comparison to uninjured fins. Another group [Nolte et al. (11)] employed LC-MS in combination with SILAC (stable isotope labeling by/with amino acids in cell culture), in which newly synthesized proteins can be identified (15), and identified more than 5,000 labeled proteins across samples. All of these studies had a different focus of research (tissue characterization, ECM composition, protein abundance alterations) and made use of different methods (labeling-based and label-free proteomics) and instruments (time-of-flight, linear ion trap, and Orbitrap mass analyzers) explaining the variance in number of identified proteins which have, however, demonstrated the variety of protein changes taking place during tissue regeneration of the fin.

Here, we made use of label-free proteomics to study the abundance of proteins in the regenerating fin during regenerative outgrowth (4 days post amputation, dpa) in normal (vehicle treated) vs. perturbed conditions after synthetic glucocorticoid exposure (50 μM prednisolone) and compared this to uninjured untreated fins. At 4 dpa, fin regenerate length does not significantly differ between vehicle and prednisolone treated zebrafish; and decreased regenerate length is only reliably detected at 7 dpa and later [Figure S1, (8)]. Furthermore, unchanged innate immune cell (macrophage) and osteoblast numbers suggest that regenerate composition is not profoundly changed at 4 dpa [Figure S1, (8)], while gene expression may be already affected. In addition to conventional differential abundance (“single protein”) analysis, we implemented functional clustering and enrichment analysis and weighted gene correlation network analysis (WGCNA) to identify affected protein groups. In our experiments, we identified almost 7,000 proteins and quantified more than 3,600 proteins in uninjured and regenerating fin tissue, out of which 303 proteins showed a highly stringent, significantly altered abundance in regenerating tissue. Expression of 103 proteins was significantly changed by glucocorticoid treatment. Differential abundance analysis, functional clustering, and WGCNA suggest that synthetic glucocorticoids lead to aberrant cell metabolism in the regenerating tissue. This is particularly true for processes that relate to ATPase coupled transport, vesicular trafficking and macromolecular transport within cells, which further emphasizes the presence of substantial inhibitory effects of glucocorticoids in fin tissue including bone.

## Results

### Sample Quality (Filtering and Partial Least Square Discriminant Analysis)

**Figure 1:**
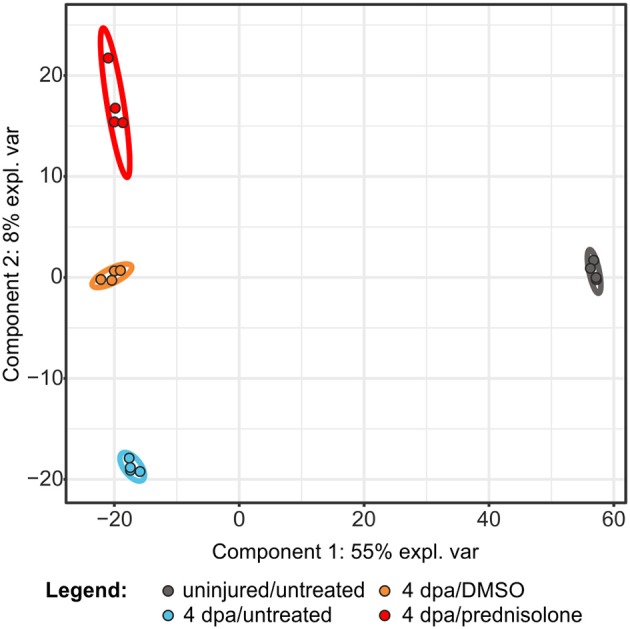
Partial least square discriminant analysis using quantitative values of all identified proteins. expl. var., explained variance.

In order to investigate the effects of systemic glucocorticoid exposure on the proteome of regenerating fins we analyzed the following samples by LC-MS: untreated uninjured fin tissue (uninjured/untreated), untreated regenerating fin tissue at 4 dpa (4 dpa/untreated), vehicle (DMSO) treated regenerating fin tissue at 4 dpa (4 dpa/DMSO) and prednisolone (50 μM) treated regenerating fin tissue at 4 dpa (4 dpa/prednisolone). For each sample 4 technical replicates were analyzed using a gel-electrophoresis LC-MS approach, which led to the identification of 6,490 proteins (Table S1). Of those proteins 3,607 (uninjured/untreated vs. 4 dpa/untreated) and 3,982 (4 dpa/DMSO vs. 4 dpa/prednisolone), respectively, were reliably quantified in three out of four replicates. Partial least square discriminant analysis revealed that uninjured samples cluster together and are distinct from the clusters comprising injured samples (Figure 1). Moreover, all treatment groups of the injured samples can be discriminated in distinct clusters. Within all samples, technical replicates clustered as expected indicating a good intra-sample reproducibility.

### Significantly Altered Protein Expression

**Figure 2:**
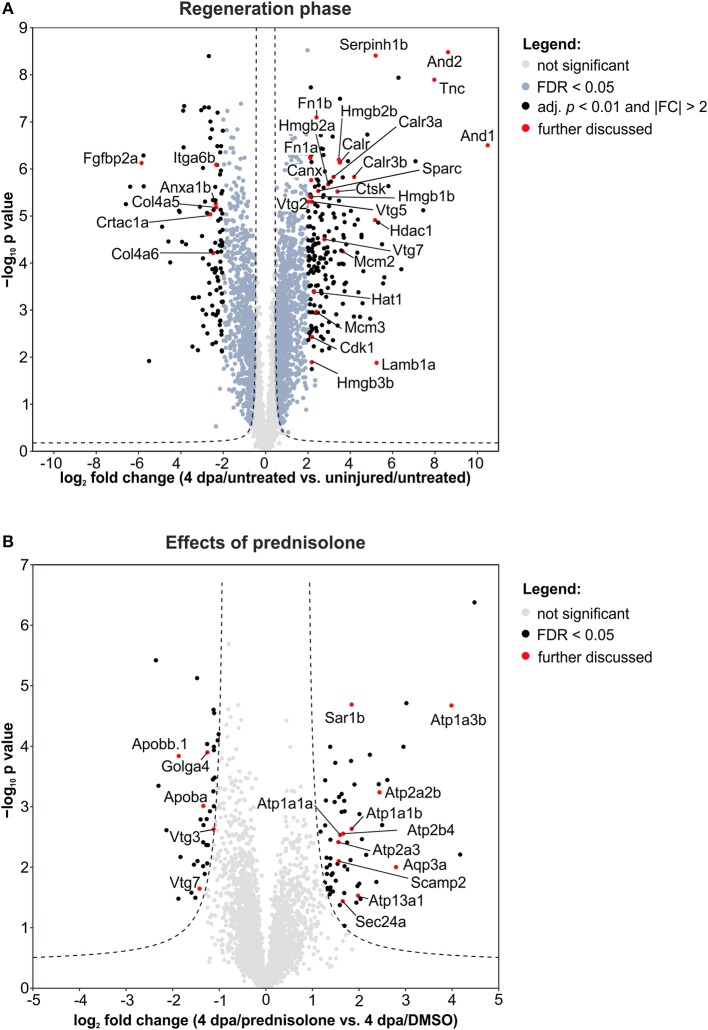
Volcano plots of all quantified proteins for the given analyses with their log_2_-transformed ratios of the mean centered abundances (fold changes, FC) and –log_10_-transformed *p* values (*t*-test, unpaired, two-sided). Dashed line represents the cut-offs from permutation-based FDR validation to identify significantly altered abundances (FDR < 0.05). Proteins highlighted in red are discussed in the text. **(A)** Proteins affected in the regeneration phase. Significantly altered proteins were further filtered by adj. *p* < 0.01 (Benjamini-Hochberg) and absolute FC > 2. **(B)** Proteins affected by the prednisolone treatment. No further filtering for significantly altered proteins was done.

Next, we employed the computational platform Perseus (16) for differential abundance analysis. Using a permutation-based FDR validation with a FDR < 0.05 as cut-off for significance proteins being affected at the regeneration stage (4 dpa/untreated vs. uninjured/untreated) or being affected by prednisolone (4 dpa/prednisolone vs. 4 dpa/DMSO) were identified. Since the above filter led to a high number of altered protein abundances at the regeneration stage and to focus the analysis on the most affected proteins, an additional stringency filter was implemented after FDR permutation for this sample set (Benjamini-Hochberg adjusted *p* < 0.01 and absolute log_2_fold change > 2, Figure 2A). Applying these criteria a significantly altered abundance for 303 proteins in regenerating fins (Figure 2A, top 20 regulated proteins in Table 1, for complete list see Table S2) and 103 proteins affected by prednisolone (Figure 2B, top 20 regulated proteins in Table 2, for complete list see Table S3) were found.

**Table 1:** Top 20 altered abundant proteins after injury.

**Accession**	**Gene name**	**Protein name**	**Adj. *p*-value**	**Log_**2**_ FC**
E7F5V5	and1	Actinodin1	2.8E-05	10.5
F6NZL0	and2	Actinodin2	3.6E-06	8.6
F1QYE2	tnc	Tenascin C	7.6E-06	8.0
A0A0R4IEM8	srsf1a	Serine/arginine-rich-splicing factor 1A	1.4E-04	7.4
Q1L8P0	crtap	Cartilage-associated protein	3.3E-05	7.1
Q6DGE4	rcn3	Reticulocalbin 3, EF-hand calcium-binding domain	8.6E-04	6.4
F1R0M8	plod1a	Procollagen-lysine, 2-oxoglutarate 5-dioxygenase 1a	7.6E-06	6.3
F1REL9	eif5	Eukaryotic translation initiation factor 5	6.0E-04	5.9
A8WIN7	plod3	Procollagen-lysine, 2-oxoglutarate 5-dioxygenase 3	7.2E-05	5.8
F1R8L1	bzw1a	Basic leucine zipper and W2 domain-containing protein 1-A	1.2E-03	5.6
A3KNH4	eif2s2	Eukaryotic translation initiation factor 2, subunit 2 beta	4.0E-04	5.5
E7F419	u2af1	U2 small nuclear RNA auxiliary factor 1	2.0E-04	5.3
Q29RF0	lamb1a	Laminin, beta 1a	2.7E-02	5.2
F1R7F7	serpinh1b	Serpin peptidase inhibitor, clade H, member 1b	3.6E-06	5.2
Q6PD83	viml	Vimentin-like	2.5E-02	−5.5
F1QLU3	dpt	Dermatopontin	7.2E-05	−5.8
F1QT60	paplna	Papilin a	3.0E-05	−5.8
F1Q8P7	fgfbp2a	Fibroblast growth factor-binding protein 2a	3.5E-05	−5.9
F1RB94	lamb1b	Laminin, beta 1b	7.2E-05	−6.4
A0A0R4IDN1	tyrp1b	Tyrosinase-related protein 1b	1.2E-04	−6.6

*Selection based on absolute fold changes (FC). Accession, gene name and protein extracted from Uniprot KB. Benjamini-Hochberg adjusted p-values from permutation-based FDR validation and Log_2_-transformed FC from 4 dpa/untreated vs. uninjured/untreated sample comparison*.

**Table 2:** Top 20 altered abundant proteins after prednisolone treatment.

**Accession**	**Gene name**	**Protein name**	***p-*value**	**Log_**2**_ FC**
Q801U4	srsf3a	Serine/arginine-rich-splicing factor 3a	4.19E-07	4.5
Q566Z4	srd5a2a	Steroid-5-alpha-reductase, alpha polypeptide 2a	6.17E-03	4.2
Q9DEU2	atp1a3b	Sodium/potassium-transporting ATPase subunit alpha	2.12E-05	4.0
A8E7G8	tm9sf3	Transmembrane 9 superfamily member	1.94E-05	3.0
B8A445	slc44a1a	Solute carrier family 44 (choline transporter), member 1a (Fragment)	1.02E-04	3.0
Q803U6	aqp3a	Aqp3 protein (Aquaporin 3)	9.94E-03	2.8
Q1L8D8	si:rp71-15k1.1	Si:rp71-15k1.1	2.78E-02	2.8
A0A0R4IR75	stt3a	STT3A, subunit of the oligosaccharyltransferase complex (catalytic)	3.61E-04	2.6
B0S5D9	tm9sf2	Transmembrane 9 superfamily member	2.01E-03	2.5
Q6ZM60	atp2a2b	Calcium-transporting ATPase	5.78E-04	2.4
U3JB26	srsf2a	Serine/arginine-rich-splicing factor 2a (Fragment)	4.24E-04	2.4
Q6TLH9	zmat2	Z2610510D14Rik (Zinc finger, matrin-type 2)	1.75E-02	2.4
Q802Y1	srsf4	Serine/arginine-rich-splicing factor 4	1.38E-04	2.2
A3KPI6	si:ch211-288g17.3	Si:ch211-288g17.3	6.25E-03	2.2
A2AW97	si:ch211-239f4.6	Si:ch211-239f4.6	3.44E-03	2.1
F1QMY2	lbr	Lamin B receptor	3.35E-02	2.0
B7ZDB5	bgnb	Biglycan	1.32E-03	2.0
E7FFW9	gig2l	Gig2-like protein DreL	2.44E-03	−2.1
A5HAK1	rnasel2	RNase 2 (Ribonuclease-like 2)	4.51E-04	−2.3
F8W5P2	hp	Haptoglobin	3.80E-06	−2.4

*Selection based on absolute fold changes (FC). Accession, gene name and protein extracted from Uniprot KB. p-values from permutation-based FDR validation and Log_2_-transformed FC from 4 dpa/prednisolone vs. 4 dpa/DMSO sample comparison*.

### Protein Expression During Regeneration

**Figure 3:**
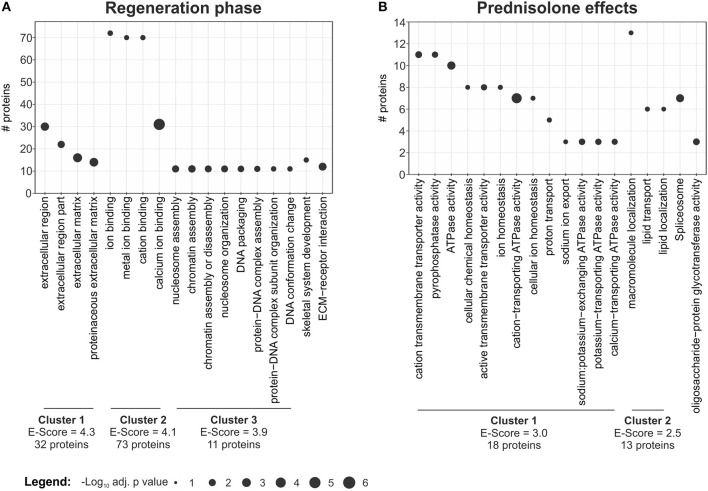
Term enrichment and clustering. Extracted enriched terms and clusters for the given analyses. Significance for enrichment (-log_10_-transformed adj. *p* value) and number of included proteins for each term are indicated. The enrichment score for each cluster was calculated using DAVID. For clustering terms were filtered for EASE < 0.05, but only terms with adj. *p* < 0.01 are reported. **(A)** Enriched terms and clusters for proteins affected in the regeneration phase. **(B)** Enriched terms and clusters for proteins affected by the prednisolone treatment.

In order to categorize proteins which are significantly regulated during regeneration (4 dpa/untreated vs. uninjured/untreated) we performed gene ontology analysis using DAVID (17). This resulted in classification of regulated proteins into three main clusters: cluster 1—extracellular region (32 non-redundant proteins, Enrichment-Score = 4.3) including the term “proteinaceous ECM,” cluster 2—ion binding (72 proteins, Enrichment-Score = 4.1), including “calcium ion binding,” and cluster 3—nucleosome assembly (11 proteins, Enrichment-Score = 3.9), including “chromatin assembly or disassembly” (Figure 3A, Table S4). In addition, proteins involved in skeletal system development (15 proteins) and proteins involved in ECM-receptor interaction (12 proteins) were identified (Figure 3A, Table S4).

We further analyzed our dataset on fin regeneration for changes in ECM composition in greater detail. In agreement with previous reports, Fibronectin 1a (Fn1a, also known as Fn1), Fibronectin 1b (Fn1b), Actinoidin1 (And1), and Actinoidin2 (And2) were enriched during regeneration (11) (Figure 2A). The same was true for Tenascin C (Tnc), a glycoprotein which is expressed in migratory cells after fin amputation (18), and Lamb1a (Laminin, beta 1a, Figure 2A) (19). On the other hand, selected ECM proteins [Collagen type IV alpha 5 (Col4a5), Collagen type IV alpha 6 (Col4a6), Cartilage acidic protein 1a (Crtac1a), Integrin alpha 6b (Itga6b)] were less abundant in regenerating fins (Figure 2A).

Within cluster 2 (ion binding proteins) several calreticulins (Calr, Calr3a, Calr3b) were induced in regenerating fins, while Annexin a1b (Anxa1b) and Calnexin (Canx) were less abundant (Figure 2A). In cluster 3, proteins such as Cyclin-dependent kinase 1 (Cdk1), DNA helicase (Mcm2 and Mcm3) and Histone acetyltransferase (Hat1) were enriched, illustrating the high proliferative activity of the regrowing tissue (Figure 2A). Hdac1 (Histone deacetylase 1), a component of the NuRD (Nucleosome Remodeling and Deacetylase) complex was also more abundant in 4 dpa/untreated fin regenerates, in agreement with a previous study (20) (Figure 2A).

Further analyses on regeneration and skeletal development-related proteins revealed that Serpinh1b [Serpin peptidase inhibitor, clade H (heat shock protein 47), member 1b], a chaperone for procollagen and known risk gene for *osteogenesis imperfecta* (type X) (21), Cathepsin k (Ctsk, a bone catabolizing enzyme), and Osteonectin (Sparc) are enriched in 4 dpa/untreated fin regenerates, in agreement with previous reports (22, 23) (Figure 2A). Furthermore, Procollagen-lysine,2-oxoglutarate 5 dioxygenase 1 (Plod1) and Plod3, enzymes which hydroxylate lysyl residues in collagen-like peptides and are thus important for the cross-linking and stabilization of collagen are enriched. At the same time, several High mobility group proteins (Hmgb1b, Hmgb2a, Hmgb2b, and Hmgb3b), which are released upon tissue damage and serve as alarmins (24), were highly abundant in regenerating fins. Notably, one of the proteins that was strongly reduced in abundance during fin regeneration, is Fibroblast growth factor-binding protein 2a (Fgfbp2a) (Figure 2A), a soluble form of the Fibroblast growth factor receptor which is implicated in regulation of Fgf signaling (25) and which is enriched proximally in uninjured zebrafish caudal fins (12).

### Protein Expression After Glucocorticoid Treatment

DAVID clustering and enrichment for proteins affected by prednisolone treatment (4 dpa/prednisolone vs. 4 dpa/DMSO) resulted in classification of regulated proteins in two clusters. Cluster 1 includes proteins that are related to “cation transmembrane transporter activity,” “ATPase activity” and alike (18 non-redundant proteins, Enrichment score 3.0, Figure 3B, Table S5). Cluster 2 includes proteins related to “macromolecule localization,” more precisely “lipid transport” and “lipid localization” (13 proteins, Enrichment score 2.5, Figure 3B, Table S5). Other regulated proteins relate to the GO terms “spliceosome” and “oligosaccharide-protein glycotransferase activity” (seven and three proteins, respectively) (Figure 3B, Table S5).

When we analyzed our dataset for the altered abundance of proteins after prednisolone exposure, we identified a variety of ATPases to be affected. In particular, the abundance of calcium-transporting ATPases both of the Sarcoplasmic reticulum Ca^2+^ ATPase (SERCA) type (Atp2a2b, Atp2a3) and of the Plasma membrane Ca^2+^ ATPase (PMCA) type (Atp2b4) were increased by the glucocorticoid (Figure 2B). Additionally, sodium/potassium-transporting ATPases (Atp1a1a, Atp1a1b, Atp1a3b) were induced upon prednisolone exposure (Figure 2B). The cation-transporting ATPase Atp13a1 was also more abundant after treatment (Figure 2B). Another protein with significantly altered abundance level upon glucocorticoid exposure was Aquaporin 3 (Aqp3a), a pore protein which allows for water passage across membranes (26) (Figure 2B). This illustrates that glucocorticoid exposure leads to profound alterations in ion transport mechanisms across membranes, which may have important implications in cell signaling and cell metabolism.

The second core theme which differed in prednisolone treated fins was lipid transport and localization. Reduced lipid transport protein abundances were found for apolipoproteins (Apoba, Apobb.1) and vitellogenins (Vtg 7, Vtg3/Phosvitinless) (Figure 2B). On the contrary, several proteins involved in vesicular trafficking were more abundant after prednisolone treatment, such as SEC24 homolog A (Sec24a) and Secretion-associated, Ras-related GTPase 1B (Sar1b, also known as Sara) (Figure 2B). Both represent components of the Coat protein complex II (CopII), which is responsible for the transport of protein and/or lipid-containing vesicles from the ER to the Golgi (26, 27). Secretory carrier-associated membrane protein 2 (Scamp2), another protein involved in vesicular transport, was induced after prednisolone treatment, too (Figure 2B). Also Golgin A4 (Golga4) showed aberrant levels in prednisolone treated fin regenerates, and was reduced (Figure 2B). Together, this indicates that vesicle transport and macromolecular localization are impaired in regenerating fins upon prednisolone exposure.

### Weighted Gene Correlation Network Analysis (WGCNA)

We implemented WGCNA to identify affected protein groups beyond the individual protein level. This network-focused approach was implemented earlier for transcriptome analysis and aims to circumvent general issues in high-throughput proteomics data, e.g., less statistical power in single protein approaches. Instead of comparing individual proteins in specific contrast, proteins are grouped in modules based on their abundance patterns across all treatment groups. These modules are then correlated to the treatments resulting in a correlation coefficient and significance for the whole protein group. As a result, key driver proteins for certain treatments and modules can be extracted by filtering proteins with a high gene significance (GS) and module membership (MM). Thus, WGCNA provides system level insights and high sensitivity to low abundances or fold changes (FC) (28).

**Figure 4:**
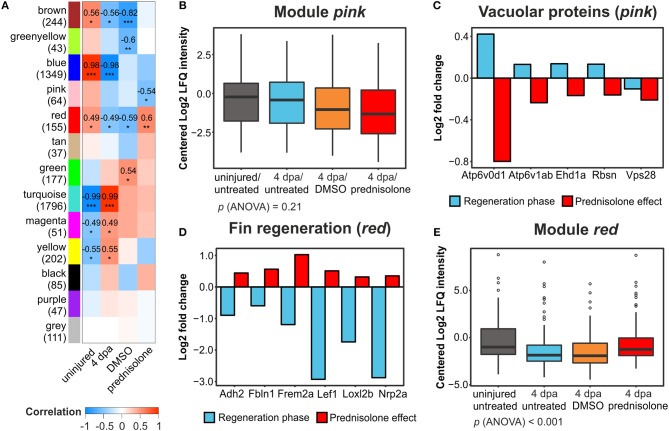
Weighted gene correlation network analysis of all filtered proteins. **(A)** Module-treatment relationship. Coefficients are indicated for |cor| > 0.4 and *p* < 0.05 with significance levels *p < 0.05, **p < 0.01, and ***p < 0.001. Numbers in brackets at the module names indicate the number of proteins in the modules. **(B)** Mean centered log_2_-transformed intensities of all proteins and treatments from the module *pink*. **(C)** Log_2_-transformed fold changes of 5 vacuolar proteins in the module *pink* identified from functional annotation analysis. **(D)** Log_2_-transformed FC of six proteins involved in fin regeneration and mesenchymal development in the module *red* identified from functional annotation analysis. **(E)** Mean centered log_2_-transformed intensities of all proteins and treatments from the module *red*.

WGCNA on our dataset categorized all identified proteins in 13 protein modules (Figure 4A, Table S1). Correlation of these modules to external traits (uninjured or 4 dpa and further treatment with DMSO or prednisolone treatment in 4 dpa trait) revealed treatment effects on the respective protein modules as a whole. Six modules (brown, blue, red, turquoise, magenta, and yellow) strongly correlated or anti-correlated to the 4 dpa trait, with outstanding coefficients of 0.99 and 0.98 and significances of *p* < 0.001 for the turquoise and blue modules, respectively (Figure 4A). These two modules include 3,145 proteins and represent the proteins affected at the regeneration stage, confirming the high number of affected proteins in the single protein analysis (Figure 2A). Term enrichment and clustering of the key drivers from those modules (GS and MM > 0.75) revealed proteins to be involved in protein synthesis (turquoise) as well as ECM composition, phospholipid and calcium binding (blue) (Table S6), again confirming single protein analysis.

Here, we focus on the effects of prednisolone. With pink (cor = −0.54, *p* < 0.05) and red (cor = 0.6, *p* < 0.01) two modules were anti-correlated and correlated to prednisolone treatment, respectively. While the mean abundance of all proteins in the pink module was similar in the uninjured/untreated and 4 dpa/untreated samples, the additional 4 dpa/DMSO and (more pronounced) the 4 dpa/prednisolone treated samples showed a reduced abundance (Figure 4B). However, ANOVA did not reveal any significant treatment-dependent alterations in this module. Nevertheless, functional enrichment and clustering analysis revealed a cluster involved in transport at the vacuolar membrane, consisting of 5 proteins (Atp6v1ab, Atpg6v0d1, Rbsn, Ehd1a, and Vps28; Table S7). Whilst abundance of four out of the five proteins was induced during regeneration, all five proteins were reduced in their abundance by prednisolone treatment (Figure 4C). None of these proteins was identified as significantly altered in their abundance in the single protein analysis, illustrating the power of WGCNA with a subsequent functional enrichment analysis.

In the red module, functional enrichment and clustering analysis revealed four clusters (Table S7): cluster 1—GTP binding (57 non-redundant proteins, Enrichment score = 2.71), cluster 2—response to organic substances (12 proteins, Enrichment score = 2.04), cluster 3—regeneration processes (12 proteins, Enrichment score = 1.62) and cellular homeostasis (11 proteins, Enrichment score = 1.59). Six proteins of cluster 3 (Frem2a, Loxl2b, Nrp2a, Cdh2, Fbln1, Lef; Figure 4D) are known to be involved in fin regeneration and/or mesenchymal cell development, which indicates that these processes are impaired by prednisolone (29–34). Furthermore, the mean abundances in the red module were reduced both in 4 dpa/untreated and 4 dpa/DMSO samples compared to uninjured/untreated and the 4 dpa/prednisolone treated groups, the latter two being very similar (treatment-group specific *p* (ANOVA) < 0.001, Figure 4E). Thus, prednisolone treatment may prevent certain proteins to shift to levels allowing successful tissue regeneration.

### *collagen 1a1b* Expression After Glucocorticoid Treatment

**Figure 5:**
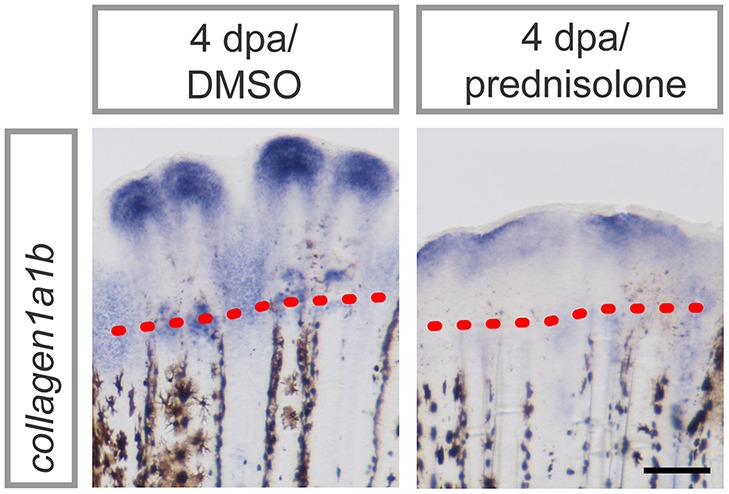
*col1a1b* expression in prednisolone treated fins at 4 dpa. Scale bar 200 μm. *n* = 4.

Applying proteomics, several ECM components were suggested to be altered in their abundance during regeneration and/or glucocorticoid treatment. To confirm altered ECM composition upon prednisolone exposure, we performed whole mount RNA *in situ* hybridization against *collagen1a1b* (*col1a1b*), a prevalent proteinaceous component of bone matrix and fibrous tissue ECM (35). *col1a1b* expression was significantly reduced after glucocorticoid exposure (Figure 5), which illustrates the sensitivity of the ECM to aberrant levels of glucocorticoids.

### Impaired Ion Transport and Lysosomal Acidification

**Figure 6:**
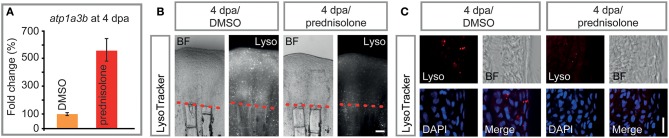
Impaired ATPase expression and lysosomal acidification. **(A)** qRT-PCR on *atp1a3b*, a sodium/potassium ATPase subunit. Mean and SD (technical error). **(B)** Live whole mount view of LysoTracker stained 4 dpa fin regenerates. Scale bar 100 μm. *n* = 5. **(C)** Longitudinal section view of fixed LysoTracker stained fin regenerates at 4 dpa. Scale bar 10 μm. BF, brightfield; Lyso, LysoTracker. *n* = 5, 5 sections minimum per fish.

Prednisolone treatment exerted strong effects on proteins that are involved in cation transmembrane transporter activity, and more specifically in ATPase activity, as detected by mass spectrometry. Thus, we tested for misexpression of selected ion-transporting ATPases in 4 dpa/prednisolone treated fin regenerates by qRT-PCR. While *atp2b4* and *atp13a1* transcripts were only mildly upregulated by prednisolone treatment (Figure S2), *atp1a3b* was about 5-fold induced (Figure 6A). This supports the idea of aberrant ATPase gene expression after prednisolone treatment.

Proteome analyses suggested a misregulation of ATPases which are involved in vesicular transport and processing of macromolecules within these. Some of these ATPases, amongst them sodium/potassium ATPases have been suggested to be involved in (the prevention of) lysosomal acidification, in addition to their role in ion-coupled transport (36). To test this, LysoTracker staining on specimens that underwent prednisolone vs. DMSO treatment for 4 days starting at the time of fin resection (i.e., the same conditions that were used in the mass spectrometry analysis) was performed. LysoTracker labels lysosomes and other acidic spherical granules within cells, and is a feasible approach to study aspects of vesicular transport and metabolism (37). Notably, prednisolone treatment strongly reduced the fluorescence resulting from LysoTracker staining in 4 dpa fin regenerates (Figure 6B). Moreover, characterization of labeling on aldehyde-fixed, cryosectioned fin tissue revealed that the amount of LysoTracker+ vesicles was strongly diminished by the glucocorticoid (Figure 6C). In particular, LysoTracker+ vesicles were significantly reduced in epidermal tissue (Figure S3A), in which about 80% of the signal was detected, while LysoTracker staining in osteoblasts and immune cells (macrophages) was generally sparse (Figures S3B,C). Our data indicate that metabolic processes that depend on acidified organelles within the cell (i.e., lysosome and autophagosome generation) are heavily misregulated by high dose synthetic glucocorticoid administration. The fact that components of the CopII complex (Sar1b, Sec24a) are misexpressed upon glucocorticoid exposure furthermore suggests that the biosynthetic-secretory pathway is affected.

## Discussion

### LC-MS to Study the Impact of Glucocorticoids on Tissue Regeneration

Here, we used fin regeneration, a model of cellular plasticity, dedifferentiation and fast regrowth of tissues including bone (38, 39), to study effects of exogenous glucocorticoid treatment. We assayed for the effects after 4 days of prednisolone exposure, at a time which is very unlikely to mainly affect direct targets of glucocorticoid signaling. Instead, our approach is intended to picture both direct and (majorly) indirect effects of the drug after a longer treatment, in order to better understand adverse effects in patients undergoing glucocorticoid therapy.

By using state of the art label-free MS technology, we identified a high number of affected proteins in distinct clusters both after injury and in combination with prednisolone treatment. To our knowledge, this study provides the most comprehensive dataset on the proteome of zebrafish fins to date. We studied the abundance of proteins, and have not addressed the question of how injury and/or glucocorticoids may alter protein activity, such as by changing phosphorylation status. In the future, it will be important to characterize the phosphoproteome of regenerating and glucocorticoid treated fin tissues to allow for a deeper understanding of metabolic alterations in adult regenerative tissues. Furthermore, a regeneration- and glucocorticoid-specific metabolome study of the fin should be performed. Notably, such holistic approaches have been carried out in other contexts, such as by Malkawi et al. (40), who demonstrated perturbations in amino acid, pyrimidine and nitrogen metabolism and lipid profile alterations in dexamethasone treated rat sera, or by Rabinowitz et al. (12), who profiled the metabolome in zebrafish to study patterning and homeostasis of uninjured adult caudal fins.

Our protein abundance-focused approach yielded interesting results. Differential expression analysis, functional clustering and WGCNA, which recently has been implemented in the proteome analysis of dexamethasone treated human bone marrow stromal cells (hBMSC) *in vitro* (41), point to profound changes in ECM composition and (calcium) ion binding capacity during regeneration, as well as altered cation transmembrane transporter activity and macromolecule localization upon prednisolone exposure. Interestingly, both injury and glucocorticoid treatment predominantly increase the abundance of sensitive proteins rather than decreasing it (197 vs. 106 proteins and 67 vs. 36 proteins, respectively). Transcriptome analysis of osteoblasts isolated from fin regenerates undergoing prednisolone treatment for 4 days shows the same trend with more genes being upregulated than downregulated (data not shown). Notably, a similar phenomenon has been observed in MC3T3-E1 and hBMSC cells undergoing dexamethasone treatment and subsequent LC-MS SILAC analysis (41, 42). This may illustrate the strong transactivational capacities of glucocorticoids in selected tissues and demonstrate increased gene expression during the course of regeneration. In combination, transactivation of genes by the glucocorticoid receptor (43) is likely to benefit from the high chromatin remodeling status of regenerating fins (20) and might add to the fact that glucocorticoids exert strong inhibitory effects in regenerating tissues such as bone and skin (8).

### Regeneration-Specific Effects at the Protein Level

Several studies have focused on the analysis of the zebrafish caudal fin proteome during development (10), adult homeostasis (12, 14), and regeneration (11, 13). Kessels et al. (10) analyzed changes in skeletal ECM composition in the head, trunk and caudal fin of zebrafish at different ontogenetic stages and identified 262 extracellular proteins, out of which many represent orthologs of mammalian proteins that are linked to bone formation. One hundred forty-four of those proteins are also present in our dataset (Table S8) including Sparc/Osteonectin, Periostin a (Postna) and biglycans (Bgna, Bgnb), which are key players in bone formation. Sing et al. (14) combined 1-DE LCMS/MS (one-dimensional gel electrophoresis followed by liquid chromatography tandem mass spectrometry) and 2-DE MALDI MS/MS (two-dimensional gel electrophoresis followed by MALDI tandem mass spectrometry) to characterize the proteome of uninjured adult caudal fin tissue and identified about 400 proteins being involved in various metabolic processes, such as catabolism and cellular component assembly. There was a moderate overlap of this study with our study (151 proteins overlap, Table S8). In both, lipid transport/localization proteins such as vitellogenins and selected apolipoproteins were found. Rabinowitz et al. (12) focused on proximally vs. distally enriched transcripts, proteins and metabolites in uninjured adult caudal fin tissue. Of the 3,061 identified proteins 2,426 were also present in our study (Table S8). Notably, besides proteins involved in skeletal system development, other transcripts involved in cation homeostasis and ion transport as well as proteins involved in lipid transport were enriched, which illustrates their abundance in homeostatic fin tissue.

To date, few studies have focused on protein analysis in regenerating caudal fin tissue. Saxena et al. (13) found 90 proteins which were differentially expressed in regenerating fins based on DIGE (difference gel electrophoresis) and subsequent mass spectrometric identification. Forty of those proteins were also identified in our study (Table S8). In contrast to our study, the data set of Saxena et al. was strongly enriched for proteins involved in cytoskeletal remodeling and cellular immune defense mechanisms. Soon after the injury neutrophils and macrophages are recruited to the resection site and into the forming regenerate where the latter are involved in the regeneration process (44, 45). At the same time cellular rearrangements occur in the stump (46), thus both detection of proteins involved in the immune response and in cytoskeletal remodeling are expected. Nevertheless, these biological processes were not strongly affected in our study. This can be explained by the use of different protein quantification techniques (fluorescence-based in-gel staining vs. label-free quantification by mass spectrometry) and the different time points of sampling of the regenerated tissue. Saxena et al. analyzed early stages of fin regeneration up to 3 dpa, while we performed our analyses on 4 dpa fin regenerates. At this later time, restoration of lost tissue including its (bony) ECM is pronounced, and ECM proteins which are involved in skeletal system development may be more prevalent. In addition, these proteins appear to underlie stronger variation than other biological processes at 4 dpa. Nevertheless, proteins such as of the Hmgb protein family, which, in addition to their nuclear, chromatin-remodeling function modulate the immune response (47) and which have been shown to be expressed after spinal cord lesion in zebrafish (48) are highly represented in our dataset.

Several ECM proteins stand out in our analysis of uninjured vs. regenerating fin samples. This is consistent with a study performed by Nolte et al. (11) who reported strong *de novo* synthesis of ECM proteins by a pulsed SILAC approach in regenerating fins. One group of detected proteins, the actinoidins (here And1, And2) are specific to fin bud mesoderm and ectoderm where they form actinotrichia in the fin (49). In the adult these non-mineralized spicules are found at the distal fin margin, and their loss (together with the loss of bony fin rays, the lepidotrichia) has been suggested to have contributed to the fin-to-limb-transition in the course of tetrapod evolution (50). Being present at the tip of the caudal fin, *and1* and *and2* transcripts and respective proteins are distally enriched during homeostasis (12) and regeneration (51, 52). In our study, aside from And1 and And2, Lamb1a was strongly enriched in 4 dpa/untreated fins. Laminins may serve a proregenerative function, being the major non-collagenous component of the basement membrane, a particular form of ECM which is found in a variety of cells (53). Specific induction of *lamb1a* takes place in the basal layer of the wound epidermis and the blastema of the fin regenerate, while it's paralogue *lamb1b* is not expressed (19, 54). The Laminin protein is deposited to the basement membrane of the basal layer of the wound epidermis in zebrafish and other teleosts, where it is likely to instruct patterning of underlying osteoblasts (19, 55, 56). In our dataset, we found that Lamb1a is induced about 36 fold, while Lamb1b is reduced more than 80 fold in 4 dpa/untreated fins, which is in agreement with the above studies. Another protein which is highly prevalent in fin regenerates at 4 dpa is Tnc, an anti-adhesive protein in the ECM of various tissues (57). Tnc, which binds to fibronectin through its FNIII repeats, thereby regulating adhesion and migration of cells (58, 59) indicates tissue rearrangement during fin regeneration. It can also bind to TLR4 and act as a danger-associated molecular pattern, thereby activating macrophages, dendritic cells and neutrophils upon tissue damage (60). Throughout fin regeneration, it is expressed in a dynamic fashion and is only weakly expressed in uninjured fins, which is why it has been suggested to be part of a transient, regeneration-specific matrix in fin mesenchyme (18, 61). In addition to Tnc, we found that both Fn1a and Fn1b protein are highly abundant in fin regenerates, in agreement with previous reports (61, 62). Both *fn1a* and *fn1b* are expressed during zebrafish heart regeneration and *fn1a* blockage by a dominant-negative inhibitor impairs this process (63). In the fin, both *fn1a* and *fn1b* are found in the blastema and also the wound epidermis of fin regenerates (61, 62). Thus, they may—like Tnc, be part of a proregenerative ECM that allows for tissue rearrangement and migration. Taken together, our data supports the idea that fin regeneration proceeds via a stage of special ECM composition, which allows for tissue rearrangement and may aid bone regeneration.

Ion-binding proteins were among the most highly regulated proteins during fin regeneration in our proteome analysis. For example, 4 dpa/untreated fins were enriched for Calr, Calr3a, and Calr3b when compared to uninjured/untreated fins. Calreticulins are calcium-binding chaperones, the function of which, to our knowledge, has not been investigated in the context of fin regeneration. However, their mRNA expression levels are significantly increased in 4 dpa regenerates (54). Notably, Calr acts as an upstream regulator of the Calcineurin complex (64), the latter controlling growth during zebrafish fin regeneration. Calcineurin inhibition leads to overgrowth of regenerating fins (65), which is brought about by misregulation of blastemal clone size (66). Furthermore, calreticulins may also inhibit the binding of nuclear hormone receptors to their response elements, which in turn acts on osteoblast gene expression such as of the differentiation factor *Osteocalcin* (67, 68). Thus, calreticulins may be instructive during zebrafish fin regeneration. Similarly, Serpinh1b (Hsp47), a chaperone important for proper folding of procollagen molecules, which was strongly upregulated in 4 dpa/untreated samples, is instructive during fin regeneration, as its knock-down affects bone forming cells and actinotrichia-formation as well as patterning (22). Another ion-binding protein whose abundance was altered during regeneration was Anxa1b. It is known that *anxa1b* levels are reduced at 4 dpa, both at the mRNA and protein level [(13, 54), this study] while other annexins such as *anxa2a/b, anxa5b*, and *anxa6* are strongly induced (69). The significance of this is to our knowledge unclear. Altogether, our data suggest that ion-binding proteins may be important in the process of fin regeneration.

### Altered Vesicle Formation Due to Glucocorticoids

In our analysis, it became obvious that prednisolone exposure leads to misexpression of several proteins which are involved in vesicular trafficking of the cell. In particular, CopII proteins such as Sec24a and Sar1b, which cover vesicles that bud off the endoplasmatic reticulum (ER) and are destined for the biosynthetic-secretory pathway (via the Golgi apparatus), are affected. Sar1b is a coat-recruitment GTPase, which is responsible for CopII coat assembly at the ER by inducing curvature and recruitment of other proteins such as Sec23 and Sec24 (70). In humans, mutations in SAR1B lead to a failure to release chylomicrons (ultra low-density lipoproteins which contain dietary lipids) into the bloodstream (71). Conversely, overexpression of SAR1B in tissue culture triggers the release of chylomicrons from human immortalized intestinal cells (72, 73). Glucocorticoids are known to increase the secretion of low-density lipoproteins, e.g., from the liver (74). In contrast, dexamethasone was shown to reduce the protein content that is secreted via matrix vesicles in hBMSC (41). Furthermore, heat shock treatment of trout, which induces high stress-levels of cortisol leads to altered exosome secretion (75). Thus, it is reasonable to assume that altered secretion of certain biomolecules takes place in glucocorticoid treated fin tissue, in which Sar1b is more abundant than in vehicle treated controls. The fact that Sec24a, another CopII protein, is highly expressed in 4 dpa/prednisolone samples points in the same direction. Mammalian Sec24 contains binding sites for membrane-spanning cargo receptors; thus its proposed function is to capture cargo in the forming vesicle which is then sent for secretion from the cell (76, 77). Remarkably, zebrafish mutants for CopII components (*sec23a* mutant *crusher* and *sec24d* mutant *bulldog*) fail to export type II Collagen and other ECM proteins in developing cartilage, which leads to skeletal defects such as a short flattened jaw (78). In medaka, mutation of *sec24d* causes malformation of the vertebrae and the skull (79). Similarly, mutations in *SEC24D* cause a syndromic form of *osteogenesis imperfecta* in humans, due to inefficient procollagen export from the ER (80). In addition to CopII proteins, Scamp2, a protein which is active in the biosynthetic-secretory pathway downstream of the Golgi apparatus (81), is affected, although in a different way than in mammalian (brain) tissues (82). Put together, this emphasizes the importance of the biosynthetic-secretory pathway during bone formation in the skeleton and suggests that this process is misregulated by glucocorticoid treatment during fin regeneration. However, whether specific cells of treated fin tissue, such as osteoblasts, are affected, or whether all cells are concerned, remains to be tested.

Besides proteins of the biosynthetic-secretory pathway, several proteins involved in transport of molecules to and from early endosomes were affected in our dataset. This, however, was only true in WGCNA which is used to identify affected protein groups rather than individual proteins. With this approach, Rbsn, Ehd1a, and Vps28 were found to be underrepresented in 4 dpa/prednisolone samples. In human cells, RBSN is a protein which is required for early endosomal fusion, by interacting with RAB5 (83). Additionally, it is involved in the transport of proteins from early endosomes to the endosomal recycling compartment and back to the plasma membrane, since its depletion leads to a retention of cargo inside of the cell (84). Likewise, EHD1 (EH domain containing 1) regulates protein recycling to the plasma membrane such as of IGF1 receptors (84, 85). Notably, corticosteroids have been shown to increase recycling of certain neurotransmitter receptors via another Rab protein, Rab4, in the rodent brain (86). Furthermore, expression of Vps28, part of the ESCRT I (endosomal sorting complex required for transport I) complex, which is important in the formation of multivesicular bodies (late endosomes which release their content into the lumen of lysosomes) (87) was mildly reduced by prednisolone treatment. Thus, WGCNA suggests that transport of proteins from early endosomes to the endosomal recycling compartment and back to the plasma membrane are affected. In particular, glucocorticoid treatment of fins might alter endosome fusion and multivesicular body formation, in addition to biomolecule secretion, a hypothesis that needs to be tested. One approach to test this could be proteome analysis of secreted vesicles, similar to a previous study, in which altered protein composition of calcifying matrix vesicles after treatment with the synthetic glucocorticoid dexamethasone was revealed in hBMSC *in vitro* (41). However, purification and enrichment of secreted vesicles from tissue is not trivial and has, to the best of our knowledge, not yet been described for adult zebrafish fin tissue. To reveal putative changes in endocytic sorting and multivesicular body formation, immunohistochemistry against Rab proteins which are either enriched in early (Rab5+) or late endosomes and multivesicular bodies (Rab7+) (88, 89) could be used in the future, in addition to Rab-based transgenic zebrafish lines which allow for *in vivo* studies of endosome biology in zebrafish (90). These and similar approaches may permit to describe the impact of high dose glucocorticoid treatment on endosome and multivesicular vesicle formation in regenerating zebrafish fins in further detail.

Another membrane-enclosed compartment of the cell, the lysosome, also seems to be affected by glucocorticoid administration. Mass spectrometry data suggested a variety of changes in ATPase expression, some of which we have confirmed at the mRNA level. In particular, *atp1a3b* expression was strongly induced in 4 dpa/prednisolone fins. This is in agreement with previous studies, in which glucocorticoid treatment led to increased sodium/potassium ATPase alpha1 subunits in cultured corneal cells (91) and patient red blood cells (92). Furthermore, next generation sequencing data on FACS isolated cells from fin regenerates suggests moderate levels of *atp1a3b* expression in osteoblasts and higher levels in macrophages (data not shown). Atp1a3b and other Atp1a subunits are part of a sodium/potassium-transporting ATPase, which regulates pH and drives the import of nutrients into animal cells by generating an (inward) sodium gradient and an (outward) potassium gradient across cell membranes (93). These and other sodium/potassium ATPases are involved in a multitude of processes, and have, for example, been suggested to promote the generation of hydroxyapatite crystals needed for bone mineralization in matrix vesicles by increasing the concentration of P_i_ (94). Notably, sodium/potassium-transporting ATPases are also a functional component of the endosomal membrane, and their suggested role here is to limit endosomal acidification (36). Their upregulation, together with the fact that levels of Atp6v1ab and Atp6v0d1, subunits of vacuolar ATPase which pumps H+ ions into the lumen of forming lysosomes, were mildly decreased by prednisolone, led us to test for the presence and extent of lysosomal acidification in regenerating fins. The amount of lysosomes in 4 dpa/prednisolone samples was strongly reduced, most prominently in the epidermis, which may have important implications for cell metabolism and signaling in the tissue. Generally, lysosomes in fin regenerates may be important to clear the tissue of damaged cells after injury. However, at 4 dpa provision of nutrients, which is achieved by the breakdown of biomolecules in the lysosome, may be more relevant to the growing tissue. Notably, inhibition of v-ATPases by bafilomycin A1 strongly impairs fin regeneration and leads to the accumulation of autophagosomes, bilayer membranous vesicles, which normally fuse with lysosomes to allow for digestion of cellular material (95). This is especially true in regions of bone formation; also in undisturbed conditions osteoblasts are very rich in autophagosomes [Figure 3 in (95)]. We did not detect high numbers of LysoTracker+ osteoblasts and macrophages with the used staining method (incubation) and their number was unchanged after prednisolone treatment. This may indicate that prednisolone exclusively impairs acidification of endosomes in the epidermis. However, we suspect that there are limitations in detection of acidified vesicles in deeper tissues of the regenerate, thus these data warrant further investigation. Notably, lysosomal acidification is required for appropriate signaling in the regenerating fin, such as of mTOR (96), and should be further investigated in different cell types of the regenerate, including osteoblasts and macrophages. Taken together, our data indicate that glucocorticoids lead to altered lysosomal acidification, via upregulation of sodium/potassium ATPase and reduction of vacuolar ATPase function, and that this process is impairing tissue restoration in the regenerating fin.

### Calcium Transport and Water Fluxes in Glucocorticoid Treated Fins

In addition to vesicular transport mechanisms, calcium transport mechanisms and osmolarity might be altered in 4 dpa/prednisolone fin samples. The abundance of calcium-transporting ATPases (both PMCA and SERCA) was increased by prednisolone. PMCA function in the regulation of cytoplasmic and extracellular Ca^2+^ levels, and one of them (*atp2b1a*) has previously been implicated in the calcification of pharyngeal teeth in zebrafish (97). Atp2b4, which was altered in our dataset, may serve a similar function, both by the export of Ca^2+^ ions and the regulation of differentiation. SERCAs, by contrast, are found in the ER which is connected to the nuclear envelope. They allow for Ca^2+^ transport from the cytosol to the lumen of the ER, a process that is tightly regulated and impacts on calcium homeostasis and signal transduction in the cell (93). Therefore, induction of the above calcium-transporting ATPases upon glucocorticoid exposure might have important implications on signaling within the fin regenerate, and may affect bone mineralization, a topic which needs to be investigated in the future.

Aquaporins are expressed tissue-specifically in mammalian and non-mammalian vertebrate species. In teleost fish, *aqp3* isoforms are expressed in gills and other epithelial tissues, and serve an osmoregulatory function (98). In adult zebrafish *aqp3a* is expressed in the eye, epidermis and gills while it is absent from muscles and brain (99). Notably, *aqp3* levels in the gills of eel are downregulated by cortisol as a reaction to adaptation from fresh- to seawater, while intestinal *aqp3* levels do not change (100). In contrast, Aqp3a levels are increased upon glucocorticoid treatment in the inner ear of mice, where they contribute to reabsorption of endolymphatic water to prevent accumulation of liquid in the inner ear (101). In 4 dpa/prednisolone treated fin regenerates Aqp3a was significantly reduced about 7 fold, and its expression was decreased at the regeneration stage. The significance of this is unclear and it remains to be tested which role *aqp3a* might play in normal fin regeneration and whether there is glucocorticoid impairment of this process. Noticably, dominant missense mutations of *aqp3a* in zebrafish lead to shortened fins (102), thus it is likely that *aqp3a* function is instructive during regeneration of the fin.

## Conclusion

Here, we present a study providing a high proteome coverage of uninjured and regenerating caudal fin tissue with and without glucocorticoid treatment. The identification of 6,940 proteins, consistent quantification of more than 3,600 proteins and two different analytic approaches allow for a better understanding of the processes underlying successful fin regeneration and the pathophysiology of glucocorticoid-induced adverse effects *in vivo*. In particular, ECM composition and ion and macromolecular transport mechanisms are affected by our manipulations, which adds novel insights to the fin regeneration process and its perturbation by glucocorticoids.

## Materials and Methods

### Animal Experiments, Fish Husbandry, and Drug Treatment

All procedures were approved by and performed in accordance with the animal handling and research regulations of the Landesdirektion Sachsen (Permit numbers AZ DD24.1-5131/354/87 and DD24.1-5131/450/4 and amendments).

Fish were bred and maintained as described (103). Fin amputations and drug treatments were performed as previously described (8, 34, 104). The treatments with 50 μM prednisolone (Sigma–Aldrich, Munich, Germany) started right after amputation. For sampling for mass spectrometry, wild type WIK zebrafish of both sexes were used. Clutch siblings were randomly assigned to the following treatment groups: uninjured/untreated, 4 dpa/untreated, 4 dpa/DMSO, and 4 dpa/prednisolone. All groups consisted of the same number of female vs. male animals from the clutches, i.e., in every group 10 females and 10 males were used (total number of animals per group 20). Uninjured/untreated and 4 dpa/untreated zebrafish, respectively, were group housed. 4 dpa/DMSO and 4 dpa/prednisolone zebrafish were drug-treated individually (8, 104). Sampling of fin tissues for whole mount RNA *in situ* hybridization was done from male wild type WIK zebrafish or male *Hsa.RUNX2-Mmu.Fos*:EGFP^zf259^ (1) in wild type WIK background. In the latter case staining for *gfp* mRNA was used as a positive control for staining (not shown). LysoTracker Red DND-99 and DND-26 (Thermo Fisher Scientific) treatments, respectively, were performed on *OlSp7:*nlsGFP^*zf*132^ zebrafish (105) and *mpeg1*:mCherry^gl23^ zebrafish (106), respectively, as previously described (96). Experiments were open, i.e., not blinded.

### qRT-PCR

Quantitative RT-PCR was performed as described (8). The following exon-spanning primers with appropriate efficiencies (1.90 < E < 2.10) were used: β*-actin* CCTTCCTGGGTATGGAATCT, GACAGCACTGTGTTGGCATA*; atp1a3b* CTGGCCGTCATTTTGGGATA, GACACACAGTGACAGTAGCC*; atp2b4* CATGCTCCTTTCTGGAACTCA, AGTCTGAGAATTCAGGCCCA; *atp13a1* TCACATTATTTCAGGCGGCA, ATTTGCCCTGAGAGGTGTAG. Relative expression was calculated using the 2^(−ΔΔC(T))^ method (107).

### Whole Mount RNA *in situ* Hybridization

Whole mount RNA *in situ* hybridizations were performed as previously described (1). *col1a1b* mRNA was detected with a previously published probe (108).

### Histology, Photography and Image Processing

Fin regenerates were fixed in 4% paraformaldehyde in phosphate buffered saline (PBS) O/N at 4°C, washes several times with PBS and transferred to 0.5 mM EDTA in PBS (pH = 7.5) for decalcification for a minimum of 12 h. Fins were incubated 30 min each in 10, 20, and 30% sucrose in PBS, followed by a minimum incubation of 30 min in 30% sucrose/embedding media (OCT, Sakura) at 1:1 and incubation O/N in embedding media at 4°C. Fins were embedded and frozen in tissue embedding media in cryomoulds and 12 μm cryosections were obtained with a Cryostat HM560 (Microm). Sections were washed in PBS and stained with DAPI (4',6-diamidino-2-phenylindole; Sigma-Aldrich, Munich).

Pictures of whole mount fin regenerates were acquired with a Leica MZ16 FA stereomicroscope equipped with a QIMAGING RETIGA-SRV camera using identical settings (magnification, contrast, gain, exposure time) and a Olympus MVX10 equipped with a DP71 camera (RNA *in situ* hybridized fins). Images of cryosections were acquired with a Zeiss Axio Imager. Respective figures in this manuscript were compiled with Adobe Photoshop CS5 extended Version 12.1 × 64 and Adobe Illustrator CS5 Version 15.1.0. Brightness and contrast have been adapted for the DAPI channel in Figure 6C (legacy option).

### Tissue Harvest and Protein Isolation for Mass Spectrometry

Fin tissue (distal half of uninjured/untreated fins, fin regenerates of 4 dpa/untreated/DMSO/prednisolone treated fins) was quickly harvested and pooled for each treatment group into cold 500 μl lysis buffer [7 M urea, 2 M thiourea, 4% CHAPS, 70 mM Dithiotreitol, 1x protease inhibitor (cOmplete^TM^ Protease inhibitor cocktail, Sigma–Aldrich, Munich, Germany), 1x phosphatase inhibitor (PhosSTOP^TM^, Sigma–Aldrich, Munich, Germany) in 50 mM Tris-HCl (pH 7.5)] into a 2 ml tube on ice using a scalpel. Samples were sonicated for 1 min using a tissue homogenizer at intervals of 10 s and gaps of 5 s on ice in the cold room to prevent samples from heating. Samples were vortexed for 30 s and snap-frozen in liquid nitrogen. Samples were thawn and vortexed for 15 min on ice in the cold room. Samples were centrifuged at 10,000 x g for 20 min at 4°C and the supernatants were transferred to new tubes, which were snap-frozen in liquid nitrogen and stored at −80°C until further use.

### Protein Fractionation and Proteolytic Cleavage

Protein concentrations were determined using Pierce 660 nm protein assay (Thermo Fisher). The sample pools were further processed in 4 technical replicates. 30 μg of each replicate were fractionated by SDS polyacrylamide gel electrophoresis using a 4% stacking and 12% resolving gel at a constant voltage of 160 V. The protein bands were stained with Coomassie and the gel was sliced in 10 fractions aiming for equal protein amounts. All fractions were reduced in 100 mM dithiothreitol, caramidomethylated in 100 mM iodoacetamide and proteolytically cleaved in-gel using trypsin (Roche, 1:50 ratio). The resulting peptides were extracted with 50% acetonitrile (ACN), 0.01% formic acid (FA) in water. The peptide samples were evaporated, reconstituted in 0.1% formic acid and stored at −20°C until measurement.

### Liquid Chromatography Mass Spectrometry (LC-MS)

4 μl of each peptide sample were injected into the UltimMate 3,000 RSLCnano system (Thermo Fisher). The peptides were desalted for 5 min at 5 ml/min [2% ACN, 0.05% trifluoroacetic acid (TFA) in water] on an Acclaim PepMap 100 (75 μm × 2 cm). Subsequently, the peptides were eluted over Acclaim PepMap 100 (75 μm × 25 cm, both columns by Thermo Fisher) using a gradient of solvent A (0.1% FA in water) and solvent B (80% ACN, 0.1% FA in water): constant 4% B for 1 min, linear increase to 30% B for 51.5 min, linear increase to 55% B for 7.5 min. Further, the column was washed with up to 99% B and reconstituted to starting conditions prior the next sample injection. The total LC runtime was 80 min per sample. The eluting peptides were electrosprayed into a QExactive HF mass spectrometer (Thermo Fisher) using a chip-based device (TriVersa NanoMate ion source, Advion) at 1.7 kV. The peptide ions were acquired in MS1 scans at a resolution (R) of 60,000, in m/z-range 350–1,550 in positive ion mode using an automated gain control (AGC) target of 3 × 106 and a max. injection time of 100 ms. The top 15 most abundant ions were selected (isolation window: 1.4 m/z) for fragmentation with higher-energy collisional dissociation (HCD) using a normalized collision energy (NCE) of 28. The fragment ions were acquired in MS2 scans at R = 15,000 using an AGC target of 2 × 105 and a max. injection time of 50 ms. Peptides were listed for dynamic exclusion for 30 s. All mass spectra were generated using Xcalibur (version 3.0).

### Protein Identification and Quantification

The acquired data were analyzed using MaxQuant (version 1.6) with the integrated search algorithm Andromeda (109). The spectra were matched to a zebrafish reference protein database (UniprotKB, 5th January 2018, 46,932 entries). The mass error was limited to 10 ppm in first search and 4.5 ppm in main search (precursor ions) as well as 20 ppm (fragment ions), respectively. A maximum of two missed cleavages was allowed for peptide identifications. Carbaminomethylation of cysteine was set as fixed modification, whereas oxidation of methionine and deamidation of asparagine and glutamine were set as variable modifications. A minimum of two inferred peptides including at least one unique peptide were required for protein inferences. Peptide spectrum matches and proteins were validated in a target-decoy approach setting the false discovery rate (FDR) < 0.05. Lable-free quantification (LFQ) intensities were automatically extracted from precursor ions and used for protein abundances. Preset parameters were used if not stated otherwise.

### Bioinformatic Analysis of Proteomics Data

Further protein analyses were conducted in Perseus (version 1.6) (16). Reverse and contaminant entries were filtered out and the remaining abundances were centered by replicate-wise median substraction. To be considered as reliably quantified proteins needed to be quantified in at least three out of four replicates of both samples of the corresponding contrast (4 dpa/untreated vs. uninjured/untreated and 4 dpa/prednisolone vs. 4 dpa/DMSO, respectively). Significantly altered abundant proteins were identified by permutation-based FDR validation setting the FDR < 0.05. To focus solely on highly affected proteins in the 4 dpa/untreated vs. uninjured/untreated contrast, proteins were further filtered by adjusted *p* < 0.01 (*t*-test, two-sided, unpaired, adjusted by Benjamini-Hochberg method) and absolute log_2_-transformed fold change (FC) > 2. Term clustering and enrichment analysis was conducted using DAVID (17, 110) and a user defined background database including all identified proteins from this study. Only gene ontology (GO) associated BP_FAT, MF_FAT and CC_FAT as well as Kyoto Encyclopedia of Genes and Genomes (KEGG) terms were analyzed for enrichment and clustering setting the EASE score < 0.05. Furthermore, for differential abundance analysis (“single protein analysis”) only terms with Benjamini-Hochberg adj. *p* < 0.05 were reported. Weighted gene correlation network analysis (WGCNA) was conducted in the R environment using the “WGCNA” package as described previously (28, 41, 111). All identified proteins were filtered to provide quantitative values in >50% of all acquired samples/replicates. Network construction and module generation was conducted with the following parameters: network type and TOM type set to unsigned, soft power = 18, deep split = 1, merge cut height = 0.4. The resulting modules were analyzed for correlations to external traits: injured or uninjured and additional treatment with prednisolone or DMSO. Key drivers were extracted by filtering on both gene significance (GS) and module membership (MM) > 0.75.

## Data Availability Statement

The mass spectrometry proteomics data have been deposited to the ProteomeXchange Consortium via the PRIDE (112) partner repository with the dataset identifier PXD013552.

## Ethics Statement

All procedures were approved by and performed in accordance with the animal handling and research regulations of the Landesdirektion Sachsen (Permit numbers AZ DD24.1-5131/354/87 and DD24.1-5131/450/4).

## Author Contributions

Experiments were designed, performed, and analyzed by JS, KG, MB, KS, and FK. JS and FK wrote the manuscript. JS, KS, and FK accept responsibility for the integrity of data analysis. All authors interpreted data and edited the manuscript.

### Conflict of Interest

The authors declare that the research was conducted in the absence of any commercial or financial relationships that could be construed as a potential conflict of interest.
