# Supplementary material for: Glucocorticoid Treatment Leads to Aberrant Ion and Macromolecular Transport in Regenerating Zebrafish Fins
Source: Front Endocrinol (Lausanne). 2019 Oct 4;10:674. doi: 10.3389/fendo.2019.00674 (PMC6787175; doi:10.3389/fendo.2019.00674)
Supplement: Supplementary file 9 [file Data_Sheet_1.pdf]

Description of Supplementary Figures and Tables

Figure S1. *mpeg*+ macrophages in 4 dpa and 14 dpa fin regenerates.

Figure S2. *atp2b4* and *atp13a1* expression levels in prednisolone treated fin regenerates (qRT-PCR).

Figure S3. LysoTracker quantification in fin regenerates.

Table S1. List of 6,490 identified proteins.

Table S2. List of 303 proteins with significantly altered protein abundance during regeneration (stringent filter).

Table S3. List of 103 proteins with significantly altered protein abundance in prednisolone treated fin regenerates.

Table S4. Gene ontology analysis (regeneration).

Table S5. Gene ontology analysis (prednisolone treatment).

Table S6. Term enrichment and clustering (WGCNA; regeneration).

Table S7. Term enrichment and clustering (WGCNA; prednisolone treatment).

Table S8. Comparison of identified proteins of this study with identified proteins of other label-free LC-MS studies on homeostatic and regenerating fins [comparison with Kessels et al. (2014) [1], Singh et al. (2011) [2], Rabinowitz et al. (2017) [3] and Saxena et al. (2012) [4]].

Figure S1

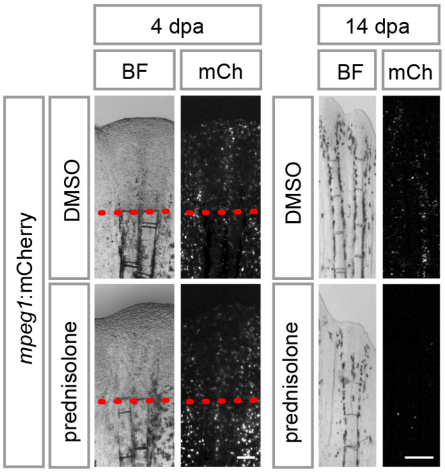

Figure S1. Macrophage number is comparable in 4 dpa/DMSO and 4 dpa/prednisolone fin regenerates in transgenic *mpeg1*:mCherry zebrafish, while it differs at 14 dpa. Scale bars 100 µm. n = 5.

Figure S2

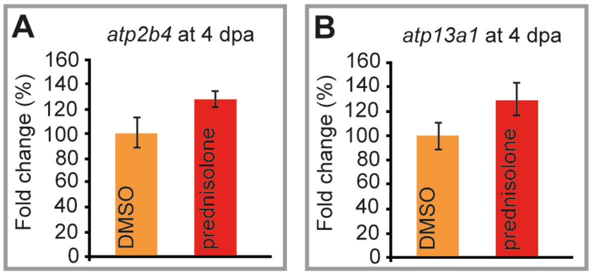

Figure S2. Mildly increased gene expression levels of ATPase subunits in 4 dpa prednisolone treated fin regenerates. **A.** qRT-PCR on *atp2b4*. **B.** qRT-PCR on *atp13a1*. Mean and SD (technical error).

Figure S3

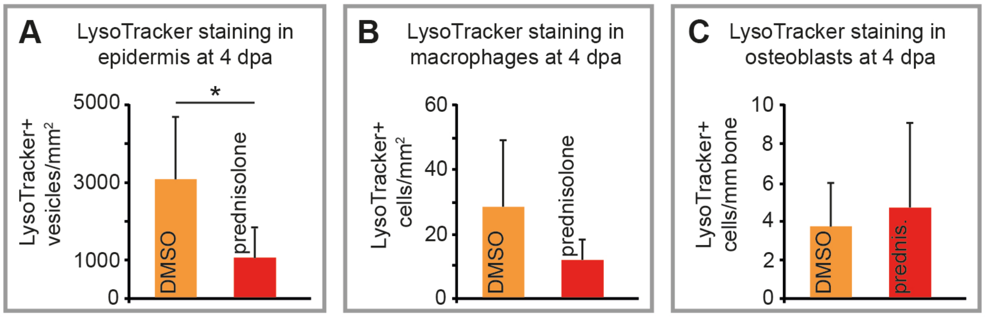

Figure S3. Quantification of LysoTracker staining in fin regenerates. **A.** Epidermis. n = 5, 5 sections minimum per fish. **B.** *mpeg1*+ macrophages. n = 5, 3 rays per fish. **C.** *osterix*+ osteoblasts. n = 5, 4 sections minimum per fish. prednis. = prednisolone. Mean and SD. Student's t-test. * $p<0.05$ (0.0357).

References in Supplement (Table S8)

[1] M.Y. Kessels, L.F. Huitema, S. Boeren, S. Kranenbarg, S. Schulte-Merker, J.L. van Leeuwen, and S.C. de Vries, Proteomics analysis of the zebrafish skeletal extracellular matrix. PLoS One 9 (2014) e90568.

[2] S.K. Singh, M.G. Lakshmi, S. Saxena, C.V. Swamy, and M.M. Idris, Proteome profile of zebrafish caudal fin based on one-dimensional gel electrophoresis LCMS/MS and two-dimensional gel electrophoresis MALDI MS/MS analysis. Journal of separation science 34 (2011) 225-32.

[3] J.S. Rabinowitz, A.M. Robitaille, Y. Wang, C.A. Ray, R. Thummel, H. Gu, D. Djukovic, D. Raftery, J.D. Berndt, and R.T. Moon, Transcriptomic, proteomic, and metabolomic landscape of positional memory in the caudal fin of zebrafish. Proc Natl Acad Sci U S A 114 (2017) E717-E726.

[4] S. Saxena, S.K. Singh, M.G. Lakshmi, V. Meghah, B. Bhatti, C.V. Swamy, C.S. Sundaram, and M.M. Idris, Proteomic analysis of zebrafish caudal fin regeneration. Mol Cell Proteomics 11 (2012) M111 014118.
